# The prevalence of thalassemia in mainland China: evidence from epidemiological surveys

**DOI:** 10.1038/s41598-017-00967-2

**Published:** 2017-04-19

**Authors:** Ketong Lai, Guifeng Huang, Li Su, Yunyan He

**Affiliations:** 1 Department of Pediatrics, the First Affiliated Hospital of Guangxi Medical University, Nanning, Guangxi China; 2 School of Public Health of Guangxi Medical University, Nanning, Guangxi China

## Abstract

Comprehensive data regarding the epidemiology and prevalence of thalassemia in mainland China are lacking. To assess the prevalence of thalassemia, we performed a meta-analysis including 16 articles published from 1981 to 2015. The overall prevalence of α-thalassemia, β-thalassemia and α + β-thalassemia was 7.88%, 2.21% and 0.48%, respectively. Trends in thalassemia prevalence in mainland China were not steady; a prevalence map based on a geographic information system (GIS) showed that the geographic distribution of thalassemia was highest in the south of China and decreased from south to north. Additionally, the most common α- and β-globin gene mutation was --^SEA^ and CD41/42, respectively. The current study provides valuable information regarding epidemiology and intervention and supports the planning, implementation and management of prevention programmes for public health.

## Introduction

Thalassemia is considered one of the most common genetic disorders in the world, with a high frequency in tropical and sub-tropical areas such as Mediterranean countries, the Indian subcontinent, the Middle East, North African and Southeast Asia^1, 2^. Thalassemia is classified into two major types, namely, α- and β-thalassemia, according to defects in these globin genes^3^. Mutations or deletions of globin genes cause abnormal haemoglobin formation, resulting in asymptomatic to severe anaemia. A foetus with α-thalassemia major will die in utero or shortly after birth, which compromises the health of the mother. β-thalassemia major patients experience severe anaemia and serious complications that include liver damage, cardiac disease^4^ and endocrine dysfunction^5^; these patients will die before 5 years of age if not treated with regular transfusions, iron chelation therapy or haematopoietic stem cell transplantation^6^. Overall, thalassemia major patients impose a considerable burden on their families and health authorities.

Presently, carrier screening, molecular diagnostics, genetic counselling, and prenatal diagnosis are employed to prevent the occurrence of thalassemia major, and these prevention programmes have had great success, leading to a decline in the birth rate of thalassemia major in some countries^7^. Surveys on thalassemia in mainland China began in the 1980s, and an understanding of the epidemiological characteristics of the disease provides important information for prevention. Previous literature indicates a high frequency of thalassemia in the population of southern China, mainly south of the Yangtze River, particularly in the provinces of Yunnan, Guangdong, Guangxi, Fujian and Sichuan. The Zeng study^8^, which was performed in many laboratories in 1987, calculated the nationwide prevalence of α-thalassemia and β-thalassemia to be 2.64% and 0.66%, respectively.

In recent years, large-scale surveys for thalassemia have been conducted in different parts of China, and the prevalence is still high^9, 10^. However, due to a lack of a comprehensive system for data collection and analysis, there are no data on the precise frequency and distribution patterns, overall burden, and trends of α- and β-thalassemia at a national scale. Furthermore, with economic improvement and population migration, thalassemia is spreading to parts of China that are north of the Yangtze River^11^. The current epidemiological characteristics of thalassemia are not completely understood. Therefore, we conducted a systematic review of recent evidence from regional population-based surveys on thalassemia to obtain a comprehensive picture of the disease in mainland China (excluding Hong Kong, Taiwan and Macao). The purpose of this study was to summarize the overall reappraisal of the prevalence of thalassemia in mainland China and to explore the epidemiological characteristics of thalassemia. The results provide a comprehensive view of thalassemia’s prevalence in China and may contribute to its control and management where it is prevalent.

## Results

### Study identification

**Figure 1:**
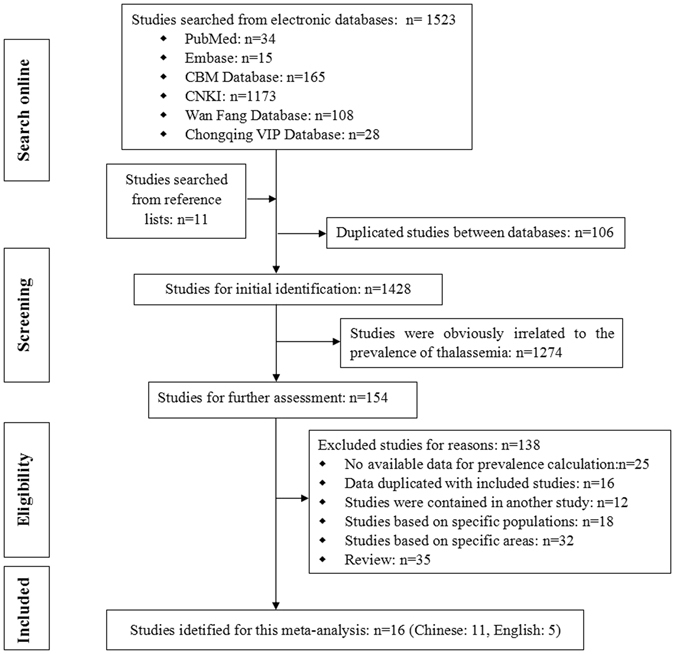
Flow chart of the selection process for the included studies.

Our preliminary literature search identified a total of 1,534 potentially relevant studies (34 on Pubmed, 15 on Embase, 165 on CBM Database, 1173 on CNKI, 108 on WanFang database, 28 on Chongqing VIP database, and the rest 11 on reference lists). After screening the titles and abstracts of these studies, we excluded 1,380 that were obvious irrelevant (n = 1274) or duplicated in the databases (n = 106). The remaining 154 studies were retrieved for a full-text assessment, and as a result, 138 were excluded because they (i) did not provide data for the prevalence calculation (n = 25), (ii) were based on special populations (n = 18) or special areas (n = 32), (iii) were duplicates (n = 16) or studies that were contained in another study (n = 12), or (iv) were reviews (n = 35). Ultimately, 16 studies were identified to be suitable for this meta-analysis^6, 8, 12–25^ (Fig. 1).

### Characteristics and quality assessment of the included studies

The general individual characteristics of the included 16 studies are summarized in Table 1. The survey dates ranged from 1981 to 2012, with an age range from 0 to 64 years. These studies covered 12 provinces (Fujian, Guangdong, Yunnan, Sichuan, Guizhou, Zhejiang, Jiangsu, Hunan, Hubei, Jiangxi, Liaoning, and Xinjiang), 1 municipality (Chongqing) and 1 autonomous region (Guangxi) of mainland China. The prevalence of α-thalassemia, β-thalassemia and α + β-thalassemia ranged from 1.20~19.87%, 0.53~6.84% and 0.08~1.22%, respectively. More details are presented in Table 1 and Supplementary Table 1 (Table S1).

**Table 1:** The characteristics of the included studies.

Study	Survey date	Location	Age range	Sample methods	Diagnostic methods	Total sample size	thalassemia [n (p)]			Quality score
							α-	β-	α + β-	
Xu *et al*.^17^	2009	Fujian	18~64y	stratified cluster random	Hb A_2_ < 2.5%for α-, Hb A_2_ > 3.5% for β- and gene analysis	11234	356 (3.17%)	148 (1.32%)	9 (0.08%)	10
Li *et al*.^22^	2008	Guangxi	12~16y	random	Hb A_2_ < 4.0%, MCV ≤ 80 for α-, Hb A_2_ > 4.0% for β- and gene analysis	1097	218 (19.87%)	50 (4.56%)	13 (1.19%)	8
Zeng *et al*.^13^	2007	Guangxi	0.5~7y	mutil-stage cluster random	Hb A_2_ < 3.5%, MCV ≤ 70 and gene analysis	2044	172 (8.41%)	—	—	9
Qiu *et al*.^19^	2006	Guangxi	3~6.5y	stratified cluster random	Hb A_2_ > 3.5% and gene analysis	2261	—	125 (5.53%)	—	9
Ma *et al*.^20^	1981	Jiangsu	—	random	MCV < 80, HbA_2_ > 3.5%	2473	—	13 (0.53%)	—	8
Cai *et al*.^24^	1999	Guangxi	0~28d	cluster	Hb Bart’s (+) and gene analysis	1028	115 (11.19%)	—	—	8
Cai *et al*.^24^	1999	Guangxi	20~44y	cluster	HbA_2_ ≥ 4.0%, MCV < 85 and gene analysis	1312	—	89 (6.78%)	16 (1.22%)	8
Chen *et al*.^23^	1999	Guangdong	0~28d	cluster random	Hb Bart’s (+) and gene analysis	1006	103 (10.24%)	—	—	7
Yao *et al*.^25^	2009	Yunnan	0~7y	stratified cluster random	Hb A_2_ < 2.5% for α-, Hb A_2_ > 3.5% for β- and gene analysis	14088	829 (5.88%)	964 (6.84%)	—	9
Liu *et al*.^21^	1998	Zhejiang	0~y	stratified cluster random	MCV < 80, HbA_2_ > 3.5%	3465	—	235 (6.78%)	—	8
Zhang *et al*.^12^	2008	Guangdong	0~28d	stratified random	Hb Bart’s (+) and gene analysis	2500	255 (10.20%)	—	—	8
Yao *et al*.^16^	2011	Chongqing	1.6~6.5y	cluster random	Hb A_2_ > 3.3% and gene analysis	1726	—	25 (1.45%)	—	8
Yu *et al*.^15^	2011	Chongqing	2~7y	cluster random	Hb A_2_ > 3.3%, MCV < 80 and gene analysis	1057	55 (5.20%)	20 (1.89%)	1 (0.09%)	9
Yin *et al*.^14^	2012	Guangdong	—	two-stage cluster random	Hb A_2_ < 3.0% for α-, Hb A_2_ > 3.5% for β- and gene analysis	26534	3531 (13.31%)	1201 (4.53%)	170 (0.64%)	8
Zeng *et al*.^8^	—	China	0~28d	random	Hb Bart’s (+) and gene analysis	12821	339 (2.64%)	—	—	8
Zeng *et al*.^8^	—	China	—	random	MCV ≤ 80, Hb A_2_ > 3.5%	361338	—	2400 (0.66%)	—	8
Xiong *et al*.^18^	2007	Guangxi	—	random	Hb A_2_ > 3.5%, MCV < 80 and gene analysis	5789	886 (15.30%)	370 (6.39%)	—	8
Pan *et al*.^6^	2000	Guangxi	0~28d	random	Hb Bart’s (+)	5400	837 (15.50%)	—	—	8
Pan *et al*.^6^	2000	Guangxi	7~40y	random	Hb A_2_ > 3.5%, MCV < 80 and gene analysis	7500	—	361 (4.81%)	—	8
Pan *et al*.^6^	2000	Guangxi	20~40y	random	Hb A_2_ > 3.5%, MCV < 80 and gene analysis	3500	—	—	11 (0.31%)	8

In Table S2, we show the detailed score of the 16 included studies. A total of five items with a maximum of 10 scores was used to analyse the quality of the identified studies. The results indicated that all the included studies were eligible; one study obtained full marks, one study obtained a score of 7, 10 studies scored an 8 and the remaining 4 studies scored a 9.

### Epidemiology of thalassemia

#### *Overall prevalence of α-thalassemia*

**Figure 2:**
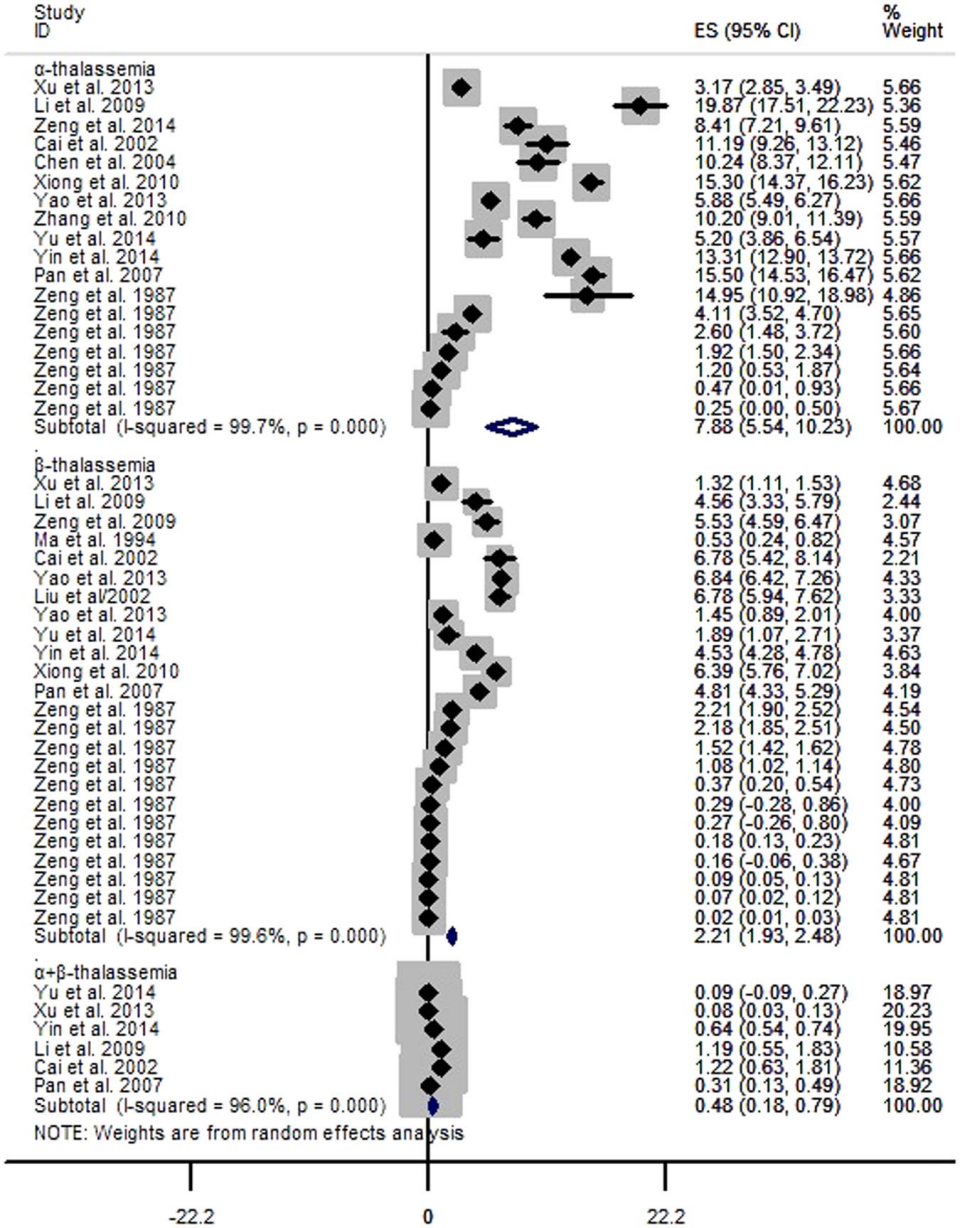
The pooled prevalence of thalassemia in mainland China.

**Figure 3:**
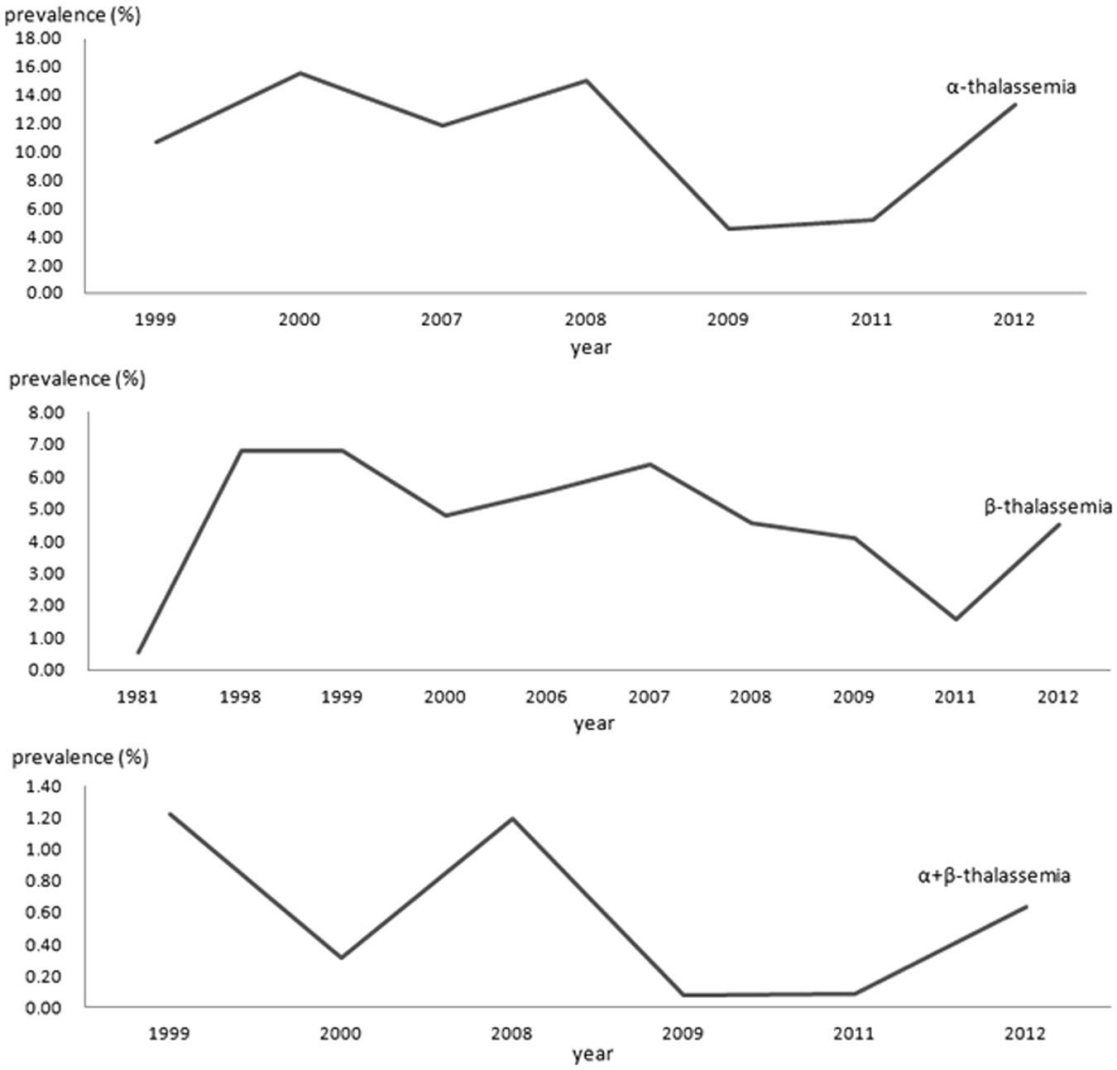
Analysis of the thalassemia prevalence by year.

**Figure 4:**
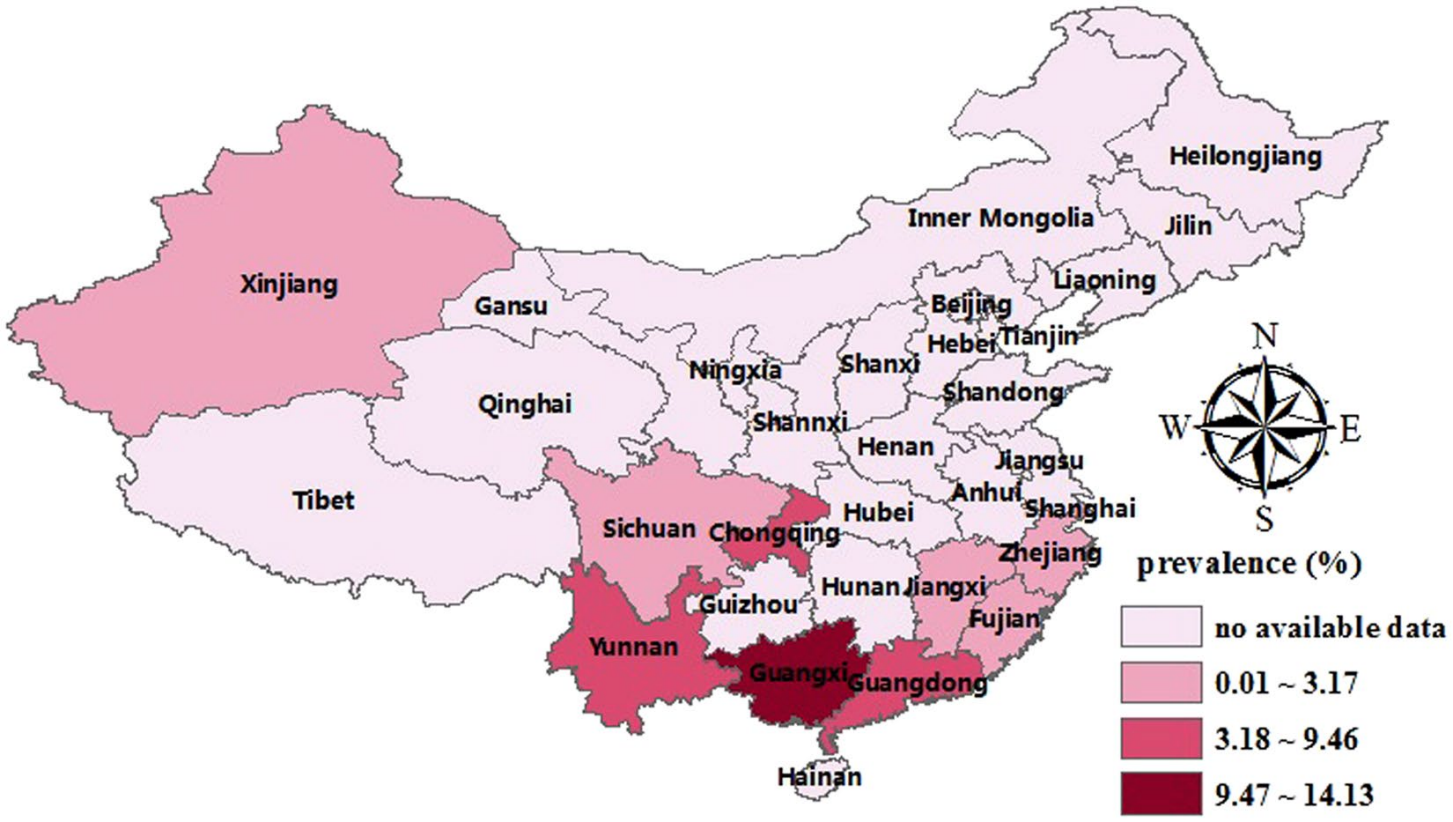
Distribution of the prevalence of α-thalassemia in different regions of mainland China.

A total of 12 studies identified 7,696 α-thalassemia cases in 84,598 subjects, with an overall prevalence of 7.88% (95%CI: 5.54~10.23) (Fig. 2, Table 2). The prevalence of α-thalassemia fluctuated annually, first declining sharply and then increasing, and this cycle continued (Fig. 3). The highest prevalence was found in Guangxi (14.13%, 95%CI: 11.12~17.13), and the second highest in Guangdong (9.46%, 95%CI: 4.00~14.92); the lowest prevalence was found in Shanghai (0.25%, 95%CI: 0.00~0.50). We analysed differences in geographical distribution across mainland China using a colour-coded map divided into four sections with different colours to indicate the highest to lowest prevalence. As shown in the map, the majority of the mainland China regions in which the epidemiologic survey was conducted were located in the south. More details are shown in Fig. 4.

**Table 2:** Summary of the prevalence estimation for thalassemia.

Types	Items		N	n	Cases	Prevalence (%)	Gene frequency (%)	95%CI	I^2^ (%)
α-thalassemia	total		12	84598	7696	7.88	—	5.54~10.23	99.7
	subtypes	--^SEA^	9	104578	2958	—	2.54	1.57~3.51	99
		-α^3.7^	9	104578	1598	—	1.59	0.93~2.24	98.8
		-α^4.2^	9	104578	554	—	0.54	0.31~0.78	96.7
		α^CS^α	8	102464	249	—	0.24	0.16~0.32	97.1
		α^WS^α	5	94308	355	—	0.26	0.17~0.36	98.9
		α^QS^α	9	104578	92	—	0.06	0.02~0.10	88.9
	diagnostic method	gene analysis	11	79198	6859	7.42	—	5.12~9.72	99.6
		no gene analysis	1	5400	837	15.50	—	14.53~16.47	99.7
	year	1991~2000	3	7434	1055	12.38	—	8.73~16.03	93.9
		2001~2010	6	36752	2716	10.36	—	6.83~13.89	99.4
		2011~	2	27591	3586	9.28	—	1.33~17.23	99.2
β-thalassemia	total		13	439874	6001	2.21	—	1.93~2.48	99.6
	subtypes	CD41/42	7	112712	931	—	0.93	0.54~1.32	98.4
		IVS-2-654	7	112712	448	—	0.25	0.11~0.40	94.8
		CD71/72	6	109260	83	—	0.07	0.03~0.12	92
		CD26	7	112712	68	—	0.07	0.03~0.10	81.9
		-28	6	109260	262	—	0.25	0.11~0.38	96.1
		CD17	7	112712	387	—	0.48	0.30~0.65	97.4
	diagnostic method	gene analysis	10	72598	3353	—	4.39	2.91~5.87	99
		no gene analysis	3	367276	2648	—	0.92	0.69~1.16	99.5
	year	1981~1990	1	2473	13	0.53	—	0.24~0.82	—
		1991~2000	3	12277	685	6.06	—	4.53~7.59	90
		2001~2010	5	34469	1657	4.92	—	1.90~7.95	99.4
		2011~	3	29317	1246	2.64	—	0.35~4.93	98.3
α+β-thalassemia	total		6	44734	220	0.48	—	0.18~0.79	96
	year	1991~2000	2	4812	27	0.72	—	−0.17~1.61	87.8
		2001~2010	2	12331	22	0.59	—	−0.50~1.67	91.2
		2011~	2	27591	171	0.37	—	−0.17~0.91	96.4

#### *Gene frequencies of α-thalassemia subtypes*

**Figure 5:**
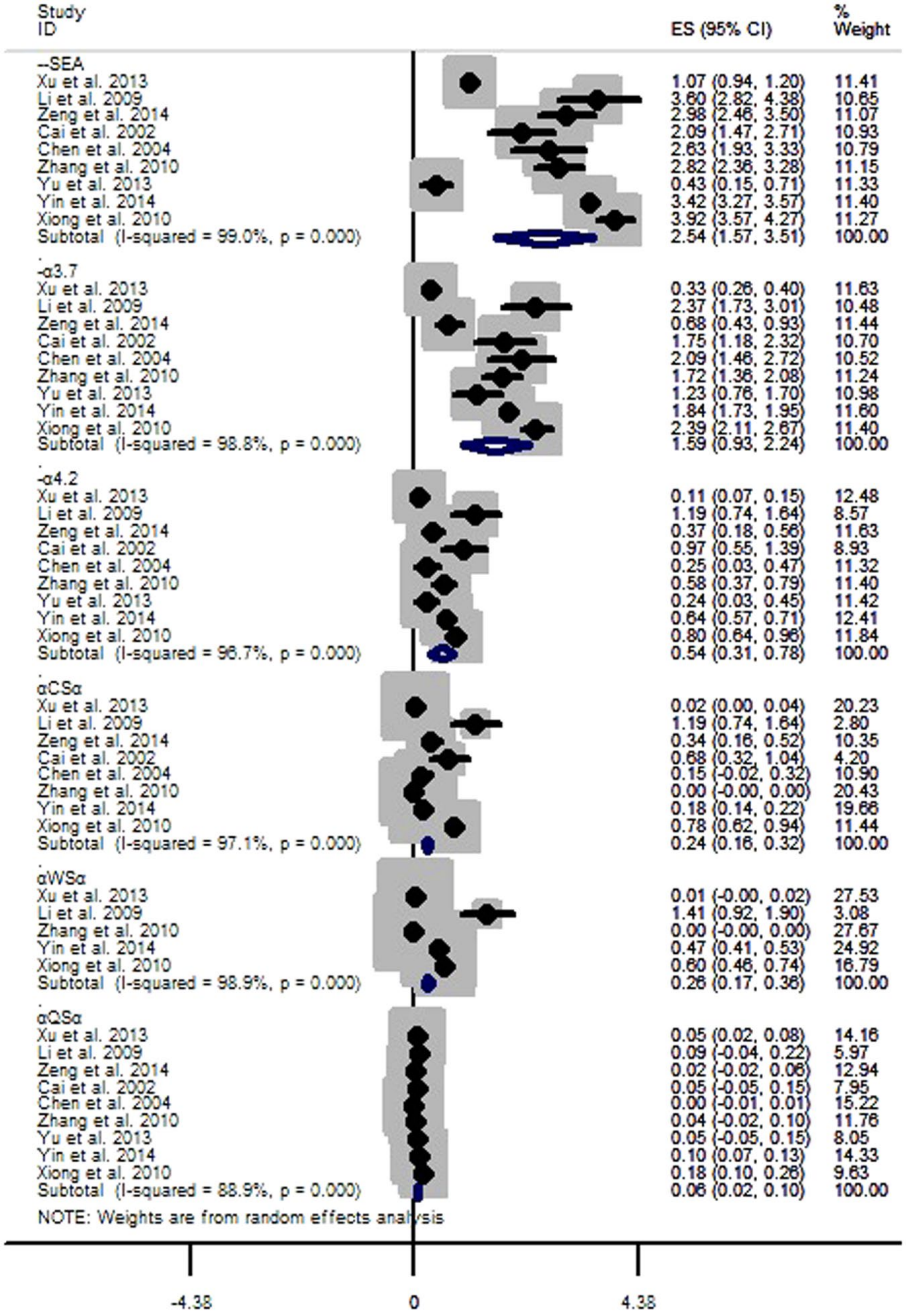
Gene frequencies of α-thalassemia subtypes.

The subtypes of α-thalassemia that we analysed in this study were --^SEA^, -α^3.7^, -α^4.2^, α^CS^α, α^WS^α and α^QS^α, and their gene frequency was 2.54% (95%CI: 1.57~3.51), 1.59% (95%CI: 0.93~2.24), 0.54% (95%CI: 0.31~0.78), 0.24% (95%CI: 0.16~0.32), 0.26% (95%CI: 0.17~0.36) and 0.06% (95%CI: 0.02~0.10), respectively (Fig. 5, Table 2).

#### *Overall prevalence of β-thalassemia*

**Figure 6:**
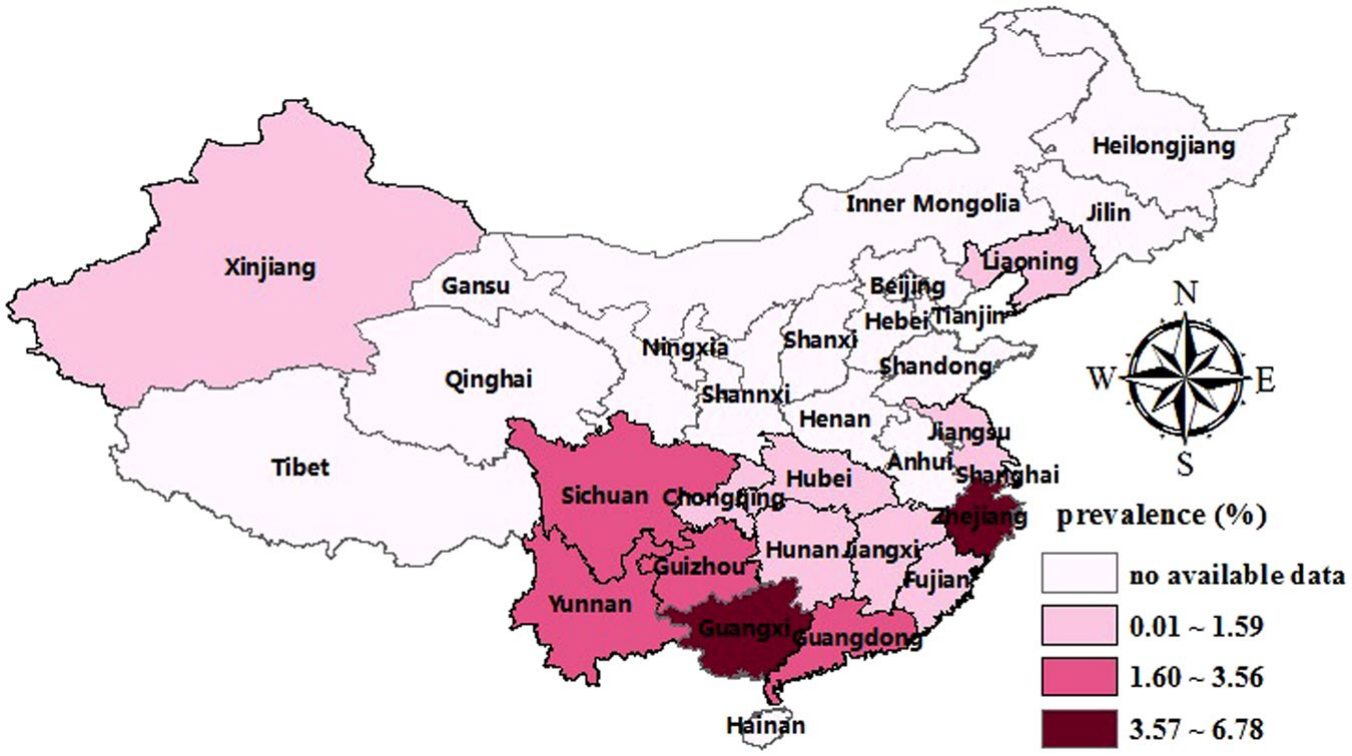
Distribution of the prevalence of β-thalassemia in different regions of mainland China.

The pooled prevalence of β-thalassemia (2.21%, 95%CI: 1.93~2.48) (Fig. 2, Table 2) was estimated on the basis of 13 related studies. Fluctuations in the prevalence were observed when we assessed the prevalence of β-thalassemia by year (Fig. 3). The highest prevalence was in Guangxi (4.91%, 95%CI: 2.67~7.15) and the lowest in Xinjiang (0.02%, 95%CI: 0.01~0.03). As shown in the colour-coded map in Fig. 6, prior epidemiological surveys of thalassemia had not been conducted in most zones.

#### *Gene frequencies of β-thalassemia subtypes*

**Figure 7:**
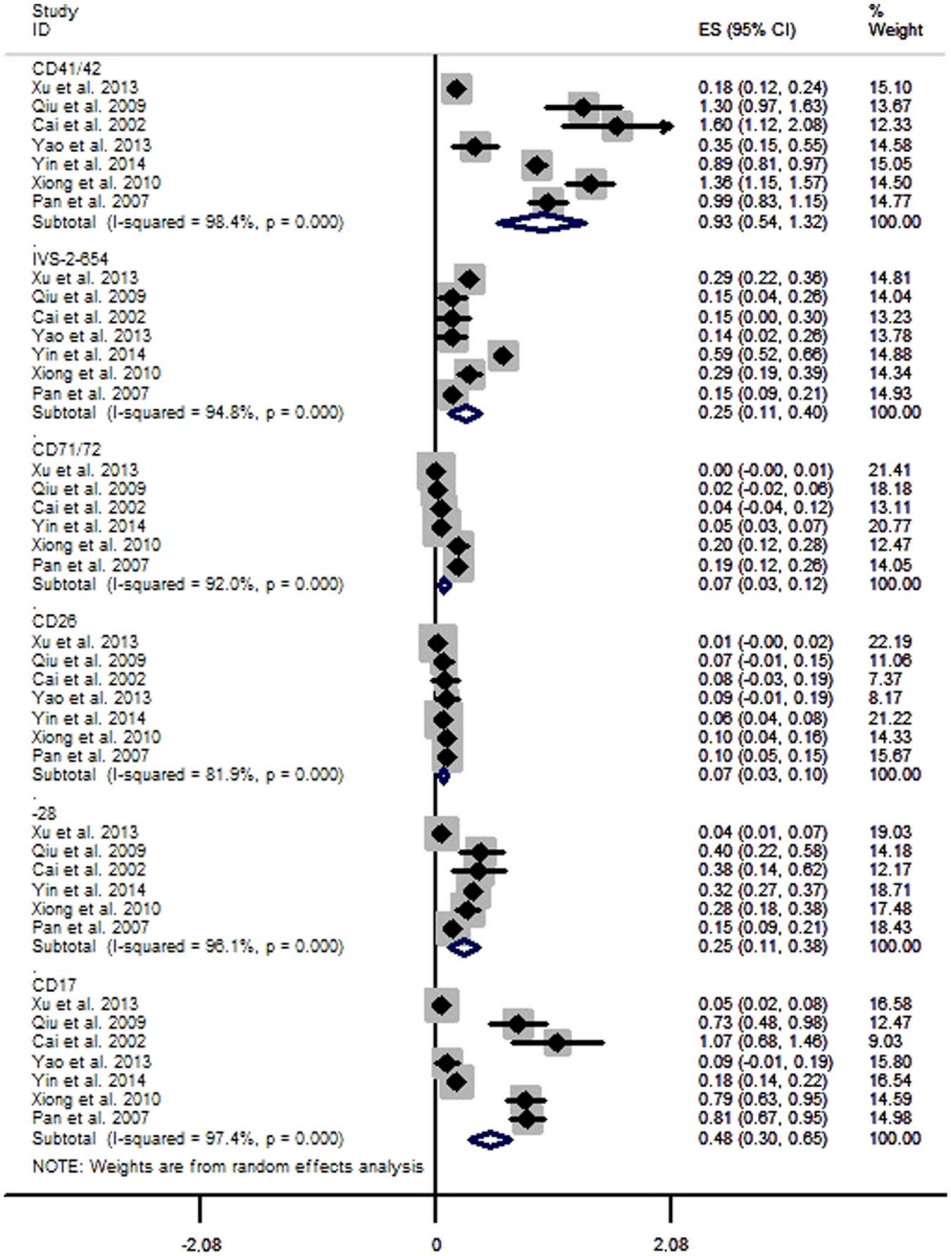
Gene frequencies of β-thalassemia subtypes.

The estimated frequencies of the six subtypes of β-thalassemia were 0.93% (95%CI: 0.54~1.32) for CD41/42, 0.25% (95%CI: 0.11~0.40) for IVS-2-654, 0.07% (95%CI: 0.03~0.12) for CD71/72, 0.07% (95%CI: 0.03~0.10) for CD26, 0.25% (95%CI: 0.11~0.38) for -28 and 0.48% (95%CI: 0.30~0.65) for CD17 (Fig. 7, Table 2).

#### *Overall prevalence of α* + *β-thalassemia*

The prevalence of α + β-thalassemia of 0.48% (Fig. 2, Table 2) was based on 6 available studies, though this prevalence exhibited large fluctuations over time (Fig. 3).

### Meta-regression

A meta-regression was performed to explore the sources of heterogeneity. In the present study, we considered several potential factors, including total sample size, quality score, diagnostic method, age range, survey date, location, and sampling method. Ultimately, none of these factors was identified as a source of heterogeneity for α-thalassemia (all *p* > 0.05). However, diagnostic method (*p* < 0.001) and survey date (NA, *p* = 0.02) were identified as a source of heterogeneity for β-thalassemia. Tables S3 and S4 present the results of this meta-regression.

### Sensitivity analysis

The pooled prevalence of α-thalassemia changed significantly when six single studies (Xu *et al*.^17^, Yao *et al*.^16^, Yin *et al*.^14^, Xiong *et al*.^18^, Pan *et al*.^6^, and Zeng *et al*.^8^) were sequentially omitted. In addition, removing the study by Zeng *et al*.^8^ contributed to the marked change in the pooled prevalence of β-thalassemia. Similarly, removing each of the studies by Xu *et al*.^17^ and Yin *et al*.^14^ altered the overall prevalence of α + β-thalassemia. More information is listed in Table S5.

### Publication bias

All three types of thalassemia presented the asymmetric shape of the funnel plots. Egger’s tests showed a significant value in α-thalassemia (*p* = 0.049), β-thalassemia (*p* < 0.001) but α + β-thalassemia (*p* = 0.27) (Table S6).

## Discussion

To the best of our knowledge, this study is the first meta-analysis of epidemiological studies on the prevalence of thalassemia in mainland China. Our results indicated that the pooled prevalence of α-, β- and α + β-thalassemia was 7.88%, 2.21% and 0.48%, respectively. The geographic distribution of thalassemia showed that the prevalence was highest in the south of China and decreased from south to north.

Thalassemia is a genetic disease for which it is possible to detect carriers using haematological indices rather than DNA analysis^26^. Increased HbA_2_ levels in peripheral venous blood is the most important feature for identifying heterozygous β-thalassemia^27^. For β-thalassemia screening, people with increased Hb A_2_ levels (HbA_2_ > 3.5%) are diagnosed with β-thalassemia^7^. Therefore, three studies regarding β-thalassemia (Zeng *et al*. in 1987^8^, Ma *et al*.^20^ and Liu *et al*.^21^) screening without gene analysis were included in our meta-analysis. In determining the prevalence of α-thalassemia, cord blood samples were quantified for Hb Bart’s when genetic analysis was not widely applied to α-thalassemia diagnosis. Pan *et al*.^6^ employed haemoglobin electrophoresis for cord blood and gene analysis simultaneously and found that cases of α-thalassemia, including heterozygous α-thalassemia, were unlikely to be missed when using a 2% cut-off of Hb Bart’s. Zeng *et al*.^8^ in which 12,821 samples of cord blood from new-borns were screened by electrophoresis, was the only study included on α-thalassemia that lacked gene analysis. Although the limitations of the experiment technology at the time prevented an assignment of thalassemia mutation subtypes, the study provides very reliable data on the nationwide incidence. Although gene analysis began to be widely used in the 2000s to ascertain thalassemia mutations in individuals, haematological indices still play a key role in diagnosis.

The 643,580 research subjects included in the 16 studies examined can generally be divided into neonates, children and adults. Although the age ranges of the different studies varied widely, subgroup analysis based on age was not performed because thalassemia is an inherited disease, and the carrying rates of different age groups are consistent in the same area. Five included studies^6, 8, 12, 23, 24^ in which the reported cases were only neonates attempted to determine the prevalence of α-thalassemia. The neonates were randomly selected such that the rates of thalassemia were representative and could be compared with the results for children or adults.

Surveys on thalassemia in China began in the 1980s. In 1987, Zeng calculated that the nationwide incidence of α-thalassemia and β-thalassemia was 2.64% and 0.66%, respectively^8^. Compared to the findings of Zeng, our meta-analysis revealed a higher prevalence of α-thalassemia (7.88%) and β-thalassemia (2.21%) in China. One explanation for the large variation in the reported prevalence is that in the previous study, a low number of samples were collected for thalassemia screening in provinces with a high incidence, such as Guangxi, where only 350 samples were collected, which made the total incidence lower. Overlooking some carriers of silent α-thalassemia mutations, such as α^WS^α/αα and α^CS^α/αα^18, 28, 29^, may be another reason. In addition, the prevalence of α + β-thalassemiain mainland China was found to be 0.48%, and our study confirmed that the double heterozygosity of α- and β-thalassemia is not rare in areas where thalassemia is common. Because there are no significant haematological differences between these double heterozygotes and β-thalassemia, it is noted to do α-thalassemia gene analysis for β-thalassemia carriers.

In our study, we generated GIS maps to provide information for 14 provincial regions and illustrate trends in the geographic distribution of thalassemia. Although the majority of the regions in which epidemiologic surveys in mainland China have been conducted are located in the south, large areas have no epidemiological survey data for thalassemia and most are located in the north. Moreover, some provinces in southern China, such as Guizhou and Hunan, have a high prevalence of β-thalassemia but scant data of α-thalassemia. With industrialization over the past 20 years and the availability of jobs in the developed areas of mainland China, many people from the southwest region have migrated to cities in the north. Indeed, population mobility and migration have resulted in a significantly increasing thalassemia prevalence on other continents, such as in Europe and North America^30, 31^. However, due to the lack of regional data in provinces in northern China with large population mobility, changes in epidemiological characteristics of thalassemia in those provinces remain unclear. Therefore, we suggest high-quality surveys should be conducted in those areas that lack data for thalassemia prevention.

Based on the present meta-analysis, the most common α-thalassemia mutationin mainland China is --^SEA^. The high gene frequency of --^SEA^ indicates that the health burden resulting from Hb H diseases and Hb Bart’s hydrops fetalis may be serious in mainland China. In addition, non-deletional α-thalassemia is not rare. α^WS^α, which is rather rare in other parts of the world^32^, is the most prevalent non-deletion type of α-thalassemia, with a gene frequency of 0.26%. Several studies on different populations have suggested that the non-deletion types of Hb H disease(--/α^T^α) are usually more severe than the deletion types (--/-α), with greater anaemia, jaundice, splenomegaly and early anaemic symptoms, and a higher proportion of patients who require blood transfusion and splenectomy^28, 33^. Therefore, the non-deletion types of α-thalassemia should be included in thalassemia prenatal diagnosis.

To explore the sources of heterogeneity, meta-regression was performed, and the diagnostic method (*p* < 0.001) and survey date (*p* = 0.02) were identified as potential sources. To mitigate heterogeneity, subgroup analysis of diagnostic methods for β-thalassemia was performed. However, heterogeneity was still high within subgroup based on diagnostic methods (Table 2). Nonetheless, it has been reported that heterogeneity cannot be avoided in a meta-analysis^34^, especially in those which based on epidemiological surveys^35^.

Our findings showed that the results were unstable when removing some individual studies one at a time. When reviewing these studies in detail, we found that the total sample size may play a role in changing the consistency of the results. A large or small sample size with a relatively higher or lower prevalence more easily caused alterations of the results. Given the limited data, we could not identify other factors to verify the robustness of the presented results.

Publication bias existed in our study, even though we comprehensively and systematically searched related studies. However, only studies published in Chinese and/or English were used, which may be a potential factor for publication bias. Insufficient data in the included studies may have also affected the results.

Several other limitations of this study should also be considered. First, because epidemiological studies on thalassemia have only been conducted in 14 provinces in China, we did not obtain good epidemiological or demographic data from all provinces. Second, the epidemiological data available mainly focuses on southern China, particularly in regions of minority nationalities, which could impact the results of our study. Third, the strategies and methodology may also have influenced the estimation of the prevalence of thalassemia. Indeed, some carriers of silent α-thalassemia mutations may not be identified by haematological indices without gene analysis. Because of these limitations, caution should be exercised in interpreting the results and in prescribing direct policy recommendations based on this meta-analysis alone. Nevertheless, our meta-analysis covered and combined most of the available epidemiologic data to generate a reasonably precise estimate of the prevalence of thalassemia.

We conducted the first meta-analysis of the prevalence of thalassemia in mainland China from 1981 to 2015, revealing the epidemiological characteristics of thalassemia. These results show that the overall prevalence of the disease is still high. Individuals in southern China have a higher risk of getting a severe form of thalassemia than those in other regions of China. In the future, epidemic research in northern China and comprehensive measures for epidemic prevention and control in southern China are needed to combat the heavy burden of thalassemia in China.

## Methods

### Study identification

We performed this meta-analysis on the basis of a systematic and comprehensive search of research on the prevalence of thalassemia in mainland China. Six electronic databases, including the Chinese National Knowledge Infrastructure database (CNKI), the WanFang database, the Chongqing VIP database, the Chinese Biological Medical Literature database (CBM), PubMed, and EMbase, were used for the identification of related studies from their establishment to January 1, 2016. The following key words were used when searching the Chinese databases: ‘thalassemia’; ‘prevalence’; and ‘epidemiology’. In addition, the key words ‘China’ and ‘meta-analysis’ were used in the English databases. We also retrieved the reference lists so that we did not overlook a related study.

### Selection criteria

We used the studies for this meta-analysis that met the following criteria:

(i) Studies were cross-sectional and conducted in mainland China (not including Hong Kong, Macao, and Taiwan);
(ii) Studies stated the prevalence of thalassemia or the related available data (the number of the participants and the number of the thalassemia patients) to calculate the prevalence of thalassemia;
(iii) Studies were published in Chinese and/or English; and
(iv) Studies were based on epidemiological surveys in general populations.

We excluded the studies that met any of the following criteria:

(i) Studies did not provide the relevant data for the prevalence of thalassemia;
(ii) Studies were based on the special populations (e.g., the elderly, women, or workers) or special areas (e.g., schools, factories, or earthquake areas); and
(iii) Duplicate studies or studies that were contained within another study.

The selection of the studies was performed by two authors independently. When the authors disagreed and could not reach an agreement after discussion, a third author was involved to reach a consensus.

### Data extraction and quality assessment of the included studies

After reaching a consensus on the included studies, the data were extracted and entered into an Excel spreadsheet, including the author, publication year, survey date, age range, location, sampling method, diagnostic method, total sample size, the number of individuals in each gender (males and females), the number of patients (including α-, β- and α + β-thalassemia), and the number and gene frequency of the subtypes (--^SEA^, -α^3.7^, -α^4.2^, α^CS^α, α^WS^α and α^QS^α of α-thalassemia; CD41/42, IVS-2-654, CD71/72, CD26, -28 and CD17 of β-thalassemia). The quality of the included studies was assessed by the 5 items and listed in the study by Li *et al*.^36^ according to the “Strengthening the Reporting of Observational Studies in Epidemiology” (STROBE) guidelines^37^. Each item was divided into 3 different levels with different scores (high risk or unclear = 0, moderate risk = 1, and low risk = 2).

Two authors finished the work independently and discussed the issues when disagreements occurred. If these authors could not reach a consensus, another author assisted in making the final decision.

### Statistical analysis

The present meta-analysis was conducted using Stata version 12.0 (Stata Corporation, College Station, TX, USA). The DeSimonian and Laird method was used to estimate prevalence, 95% confidence intervals (95%CI), and the proportion of α- or β-thalassemia subtypes. Prevalence is expressed as a percentage; if the number of the thalassemia patients was 0, we assigned a value of ‘0.01’ to retain all useful data when conducting calculations. Additionally, for the study conducted in multiple regions of mainland China, the data for each region were extracted independently for later analysis. For example, Zeng *et al*. conducted a multi-region study in mainland China, and we extracted the available data in corresponding single regions. ESRI ArcGIS 10.0 version for desktop (http://www.esri.com/software/arcgis/arcgis-for-desktop) was used to assess differences in geographic distribution. Heterogeneity was analysed using Cochran’s *x* ^2^-based Q test and I^2^ statistics (which ranged from 0 to 100%). Heterogeneity was considered to be moderate or high at *p* < 0.1 or I^2^ ≧ 50%, and a random-effects model (the DeSimonian and Laird method) was selected for the meta-analysis. Otherwise, a Mantel-Haenszel fixed-effects model was used. Meta-regression was conducted to analyse the sources of heterogeneity. Sensitivity analysis was performed to assess the effects of single study on the consistency of the results after excluding the included studies sequentially. To evaluate publication bias, funnel plots and an Egger’s test were used.

## Electronic supplementary material

Supplementary Information
