# Supplementary material for: The prevalence of thalassemia in mainland China: evidence from epidemiological surveys
Source: Sci Rep. 2017 Apr 19;7:920. doi: 10.1038/s41598-017-00967-2 (PMC5430438; doi:10.1038/s41598-017-00967-2)
Supplement: Supplementary file 1 — Supplementary Information [file 41598_2017_967_MOESM1_ESM.pdf]

Supplement Information

The prevalence of thalassemia in mainland China: evidence from epidemiological surveys

Ketong Lai[1], Guifeng Huang[2], Li Su[2], Yunyan He[1,*]

[1]Department of Pediatrics,the First Affiliated Hospital of Guangxi Medical University, Nanning, Guangxi, China.

[2] School of Public Health of Guangxi Medical University, Nanning, Guangxi, China.

*Correspondence and requests for materials should be addressed to Y.H. (email: yunyanhe@aliyun.com)

Table S1. The characteristics of the included studies

		Gene frequency of α-thalassemia subtype [n (%)]						Gene frequency of β-thalassemia subtype [n (%)]					
Study	Survey date	$--^{SEA}$	$-\alpha^{3.7}$	$-\alpha^{4.2}$	$\alpha^{CS}\alpha$	$\alpha^{WS}\alpha$	$\alpha^{QS}\alpha$	CD41/42	IVS-2-654	CD71/72	CD26 (βE)	-28	CD17
Xu et al. 2013	2009	240 (1.07)	75 (0.33)	24 (0.12)	5 (0.02)	2 (0.01)	12 (0.05)	40 (0.18)	65 (0.29)	1 (0.004)	2 (0.01)	10 (0.05)	12 (0.05)
Li et al. 2009	2008	79 (3.60)	52 (2.37)	26 (1.19)	26 (1.19)	31 (1.41)	2 (0.09)	-	-	-	-	-	-
Zeng et al. 2014	2007	122 (2.98)	28 (0.68)	15 (0.37)	14（0.34）	-	1（0.02）	-	-	-	-	-	-
Qiu et al. 2009	2006	-	-	-	-	-	-	59 (1.30)	7 (0.15)	1 (0.02)	3 (0.07)	18 (0.40)	33 (0.73)
Cai et al. 2002	1999	43 (2.09)	36 (1.75)	20 (0.97)	14（0.68）		1（0.05）	42 (1.6)	4 (0.15)	1 (0.04)	2 (0.08)	10 (0.38)	28 (1.07)
Chen et al. 2004	1999	53 (2.63)	42 (2.09)	5 (0.25)	3（0.15）		0（0）	-	-	-	-	-	-
Zhang et al. 2010	2008	141(2.82)	86 (1.72)	29 (0.58)	0 (0)	0 (0)	2 (0.04)	-	-	-	-	-	-
Yao et al. 2013	2011	-	-	-	-	-	-	12 (0.35)	5 (0.14)	-	3 (0/087)	-	3 (0.087)
Yu et al. 2013	2011	9 (0.43)	26 (1.23)	5 (0.24)	-	-	1(0.05)	-	-	-	-	-	-
Yin et al. 2014	2012	1817 (3.42)	976 (1.84)	337 (0.64)	97(0.18)	252(0.47)	52(0.1)	471 (0.89)	312 (0.59)	29 (0.05)	31(0.06)	170 (0.32)	98 (0.18)
Pan et al. 2007	2000	-	-	-	-	-	-	149 (1.00)	22 (0.15)	28 (0.19)	15 (0.10)	22 (0.15)	121 (0.81)

Table S2. Quality score assessment of included studies

Study	Survey year	Province	Quality score assessment					Total score
			Sample population	Sample size	Participation rate	Outcome assessment	Analytical methods to control for bias	
Xu et al. 2013	2009	Fujian	2	2	2	2	2	10
Li et al. 2009	2008	Guangxi	1	1	2	2	2	8
Zeng et al. 2014	2007	Guangxi	2	1	2	2	2	9
Qiu et al. 2009	2006	Guangxi	2	1	2	2	2	9
Ma et al. 1994	1981	Jiangsu	1	1	2	2	2	8
Cai et al. 2002	1999	Guangxi	1	1	2	2	2	8
Chen et al. 2004	1999	Guangdong	1	1	2	1	2	7
Yao et al. 2013	2009	Yunnan	2	2	2	2	1	9
Liu et al/2002	1998	Zhejiang	1	1	2	2	2	8
Zhang et al. 2010	2008	Guangdong	1	1	2	2	2	8
Yao et al. 2013	2011	Chongqing	1	1	2	2	2	8
Yu et al. 2013	2011	Chongqing	1	2	2	2	2	9
Yin et al. 2014	2012	Guangdong	1	1	2	2	2	8
Zeng et al. 1987	-	China	1	1	2	2	2	8
Xiong et al. 2010	2007	Guangxi	1	1	2	2	2	8
Pan et al. 2007	2000	Guangxi	1	1	2	2	2	8

Table S3. Meta-regression results of α-thalassemia

	Covariates	N*	Coef. (95%CI)	P-value
total sample size	-	18	0.00(-0.00~-0.00)	0.56
quality score	10(Ref.)	1		
	7	1	7.07(-12.54~26.68)	0.45
	8	13	5.28(-9.04~19.60)	0.44
	9	3	3.33(-12.61~19.27)	0.66
diagnostic method	no gene analysis(Ref.)	17		
	gene analysis	1	-8.04(-20.92~4.83)	0.2
age range	2~7y(Ref.)	1		
	12~16y	1	14.67(-2.86~32.21)	0.09
	0.5~7y	11	3.21(-14.17~20.59)	0.69
	0~28d	1	1.28(-11.57~14.13)	0.83
	0~7y	1	0.68(-16.66~18.02)	0.93
	18~64y	1	-2.03(-19.36~15.31)	0.8
	NA	2	9.10(-5.94~24.14)	0.21
survey date	2012(Ref.)	1		
	1999	2	-2.59(-15.15~9.96)	0.65
	2000	1	2.19(-12.23~16.61)	0.74
	2007	2	-1.44(-13.93~11.04)	0.8
	2008	2	1.59(-10.95~14.14)	0.78
	2009	2	-8.78(-21.24~3.67)	0.15
	2011	1	-8.11(-22.57~6.35)	0.24
	NA	7	-9.96(-20.86~0.93)	0.07
Location	Chongqing(Ref.)	1		
	Fujian	1	-2.03(-14.77~10.71)	0.72
	Guangdong	4	-4.25(-5.89~14.39)	0.36
	Guangxi	6	8.93(-0.90~18.77)	0.07
	Jiangxi	1	-2.60(-15.41~10.21)	0.65
	Shanghai	1	-4.95(-17.69~7.79)	0.4
	Sichuan	1	-3.28(-16.03~9.47)	0.57
	Xinjiang	1	-4.73(-17.48~8.02)	0.42
	Yunnan	1	0.68(-12.07~13.43)	0.91
	Zhijiang	1	-4.00(16.76~8.76)	0.49
sample methods	two-stage cluster random(Ref.)	1		
	random	1	6.56(-13.10~26.22)	0.47
	stratified cluster random	2	-8.79(-25.66~8.09)	0.27
	NA	9	-7.16(-21.69~7.37)	0.3
	cluster	1	-2.12(-21.72~17.48)	0.81
	cluster random	2	-5.60(-22.52~11.31)	0.48
	multi-stage cluster random	1	-4.90(-24.43~14.63)	0.59
	stratified random	1	-3.11(-22.63~16.41)	0.73

N: number of studies;Coef. : Regression coefficient; Ref. : Reference category; NA: not available.

Table S4. Meta-regression results of β-thalassemia

	Covariates	N*	Coef. (95%CI)	P-value
total sample size	-	24	-0.00(-0.00~0.00)	0.19
quality score	9(Ref.)	3		
	8	20	-2.59(-5.76~0.59)	0.11
	10	1	-3.44(-9.32~2.43)	0.24
diagnostic method	no gene analysis(Ref.)	14		
	gene analysis	10	3.27(1.61~4.94)	0
age range	7~40y(Ref.)	1		
	12~16y	1	-0.25(-6.04~5.54)	0.93
	0~y	1	1.97(-3.74~7.68)	0.47
	0~7y	1	2.03(-3.62~7.68)	0.45
	1.6~6.5y	1	-3.36(-9.03~2.30)	0.22
	18~64y	1	-3.49(-9.13~2.15)	0.21
	20~44y	1	1.97(-3.86~7.80)	0.48
	2~7y	1	-2.92(-8.63~2.79)	0.29
	3~6.5y	1	0.72(-35.01~6.45)	0.79
	NA	15	-3.49(-7.62~0.64)	0.09
survey date	2012(Ref.)	1		
	1981	1	-4.00(-8.05~-0.46)	0.052
	1998	1	2.25(-1.89~6.39)	0.26
	1999	1	2.25(-2.05~6.55)	0.28
	2000	1	0.28(-3.79~4.35)	0.88
	2006	1	1.00(-3.17~5.17)	0.61
	2007	1	1.86(-2.23~5.95)	0.34
	2008	1	0.03(-4.23~4.29)	0.99
	2009	2	-0.47(-3.98~3.03)	0.77
	2011	2	-2.87(-6.40~0.67)	0.1
	NA	12	-3.83(-6.80~0.85)	0.02
location	Zhejiang(Ref.)	1		
	Fujian	2	-6.04(-12.38~0.30)	0.06
	Guangdong	2	-3.98(-10.31~-2.36)	0.19
	Guangxi	6	-1.88(-7.50~3.74)	0.47
	Guizhou	1	-4.57(-11.87~2.73)	0.19
	Hubei	1	-6.69(-13.98~0.60)	0.07
	Hunan	1	-6.41(-13.71~0.89)	0.08
	Jiangsu	1	-6.25(-13.55~1.05)	0.09
	Jiangxi	1	-6.60(-13.89~0.69)	0.07
	Liaoning	1	-6.49(-13.81~0.83)	0.08
	Shanghai	1	-6.71(-14.00~0.58)	0.07
	Sichuan	1	-4.60(-11.90~2.70)	0.19
	Xinjiang	1	-6.76(-14.05~0.53)	0.07
	Yunnan	2	-3.22(-9.56~3.13)	0.28
	Chongqing	2	-5.11(-11.47~-1.25)	0.1
sample methods	two-stage cluster random(Ref.)	1		
	cluster random	2	-2.86(-8.22~2.49)	0.28
	stratified cluster random	4	0.56(-4.32~5.44)	0.81
	NA	14	-3.14(-7.64~1.37)	0.16
	cluster	1	2.25(-4.07~8.57)	0.46
	ramdom	2	-2.07(-7.44~3.30)	0.43

N: number of studies;Coef. : Regression coefficient; Ref. : Reference category; NA: not available.

Table S5. The results of sensitivity analysis

Diseases	Factors	Location	Age range	Total sample size	Prevalence (%)	Estimate	95%CI
α-thalassemia	Overall	-	-	84598	7.88	-	-
	Combined	-	-	-	-	4.07	3.94~4.20
	Xu et al. 2013	Fujian	18~64y	11234	3.17	4.24	4.10~4.38
	Yao et al. 2013	Yunnan	0~7y	14088	5.88	3.84	3.70~3.98
	Yin et al. 2014	Guangdong	-	26534	13.31	3.04	2.90~3.18
	Xiong et al. 2010	Guangxi	-	5789	15.30	3.84	3.71~3.98
	Pan et al. 2007	Guangxi	0~28d	5400	1.20	3.86	3.73~3.99
	Zeng et al. 1987	Sichuan	0~28d	4007	1.92	4.29	4.15~4.42
	Zeng et al. 1987	Xinjiang	0~28d	859	0.47	4.38	4.24~4.51
	Zeng et al. 1987	Shanghai	0~28d	1575	0.25	5.52	5.37~5.67
β-thalassemia	Overall	-	-	439874	2.21	-	-
	Combined	-	-	-	-	0.067	0.059~0.074
	Zeng et al. 1987	Guangdong	-	102356	1.08	0.052	0.044~0.060
	Zeng et al. 1987	Xinjiang	-	117951	0.02	0.42	0.40~0.44
α+β-thalassemia	Overall	-	-	44734	0.48	-	-
	Combined	-	-	-	-	0.22	0.17~0.26
	Xu et al. 2013	Fujian	18~64y	11234	0.08	0.50	0.43~0.58
	Yin et al. 2014	Guangdong	-	26534	0.64	0.11	0.06~0.16

Table S6. The results of publication bias

Diseases	Numer of studies	Prevalence (%)	95%CI	Heterogeneity (%)	Funnel plot	Egger's test	
						p value	95%CI
α-thalassemia	18	7.88	5.54~10.23	99.7	asymmetry	0.049	0.0448~27.476
β-thalassemia	24	2.21	1.93~2.48	99.6	asymmetry	0.000	7.889~17.943
α+β-thalassemia	6	0.48	0.18~0.79	96.0	asymmetry	0.268	-4.455~12.131
